# Finding food: how generalist predators use contact-chemosensory information to guide prey preferences

**DOI:** 10.1242/jeb.247523

**Published:** 2024-10-07

**Authors:** Richard K. Zimmer, Graham A. Ferrier, Cheryl A. Zimmer

**Affiliations:** ^1^ Department of Ecology and Evolutionary Biology, University of California, Los Angeles, Los Angeles, CA 90095, USA; ^2^ Neuroscience Program, University of California, Los Angeles, Los Angeles, CA 90095, USA; ^3^ Moreton Bay Research Station, Centre for Marine Science, and School of the Environment, University of Queensland, St Lucia, Brisbane 4072, Queensland, Australia

**Keywords:** Sensory biology, Chemoreception, Chemical cue, Quality coding, Glycoprotein, KEYSTONEin, MULTIFUNCin, Mussel, Barnacle, Sea star, Whelk

## Abstract

Understanding the processes that guide carnivores in finding and selecting prey is a fundamental, unresolved challenge in sensory biology. To our knowledge, no published work has yet revealed the complete structural identities of compounds that cue preferences by generalist predators for different prey species. With this research imperative in mind, we determined the chemistry driving consumer preferences for live intact prey using two generalist predatory species (sea stars, *Pisaster ochraceus*; whelks, *Acanthinucella spirata*), along with two foundation prey species (mussels, *Mytilus californianus*; barnacles, *Balanus glandula*), inhabiting rocky, wave-swept shores. Each prey species is known to secrete either a 29.6 kDa (named ‘KEYSTONEin’) or a 199.6 kDa (named ‘MULTIFUNCin’) glycoprotein as a contact-chemical cue. Here, experimental manipulations utilized faux prey consisting of cleaned barnacle or mussel shells infused with KEYSTONEin, MULTIFUNCin or seawater (control) gels. Whelks exhibited a strong penchant for MULTIFUNCin over KEYSTONEin, irrespective of shell type. In contrast, sea stars generally preferred KEYSTONEin over MULTIFUNCin, but this preference shifted depending on the experimental context in which they encountered physical (shell) and chemical (glycoprotein) stimuli. This study ultimately demonstrates clear and contrasting chemical preferences between sea stars and whelks. It highlights the importance of experimental setting in determining chemical preferences. Finally, it shows that prey preferences by these predators hinge only on one or two contact-protein cues, without the need for quality coding via fluid-borne compounds, low-molecular-weight substances or mixture blends.

## Teaser

**Summary:** Prey preferences by generalist predators can hinge on only one or two contact-protein cues, without the need for fluid-borne (air or water) compounds, low-molecular-weight substances, or mixture blends. Environmental setting is important in determining these chemical preferences.

## INTRODUCTION

Nearly all animals have a chemical sense as a means to exploit valuable resources and to ward off impending danger. To date, the focus of much physiological and behavioral research has emphasized the impact of fluid-borne (air and water) compounds as either attractants, deterrents, stimulants or suppressants. Particular attention has been given to the roles of chemical mixtures in cueing predator–prey interactions (Apfelbach et al., 2015; Kamio and Derby, 2016). Notably, the carnivore-derived 2-phenylethylamine stands as a pivotal component within the blend of predator odors, eliciting hard-wired aversion circuits in the rodent brain (Ferrero et al., 2011). Moreover, interactions between herbivore pheromones and host plant compounds are significant determinants of behavioral plasticity in higher-order consumers (Raffa et al., 2007), facilitating predator tracking and capturing of herbivorous prey within natural plant foliages (Erbilgin and Raffa, 2001).

The roles played by surface-adsorbed compounds as cues in locating prey have remained less explored. Important exceptions in terrestrial organisms include studies on jumping spiders (Humbel et al., 2021), insects (Lang and Menzel, 2011; Binz et al., 2016; Xue et al., 2018), birds (Skelhorn and Rowe, 2006) and snakes (Smargiassi et al., 2012; Saviola et al., 2013). Thamnophiine (non-venomous) snakes, for instance, exhibit strike-attack responses to parvalbumins and parvalbumin-like proteins in the mucus of prey fish and amphibians (Leroy et al., 2006; Smargiassi et al., 2013). In contrast, viperid (venomous) snakes employ a unique strike-and-release attack mechanism, with free disintegrins in venoms serving as molecular cues for effective relocation of envenomated prey (Saviola et al., 2013). Several species of bird and insect predators display robust attack reactions to hydrocarbons, particularly *n*-alkanes, and secondary metabolites present on the cuticular surfaces of their insect prey (Skelhorn and Rowe, 2006; Blomquist and Bagnères, 2010; Koedam et al., 2011; Xue et al., 2018).

Compared with their terrestrial counterparts, aquatic organisms have remained enigmatic in terms of the chemical identities of prey attractants and stimulants. Recent reviews (Hay, 2009; Kamio and Derby, 2016; Bornancin et al., 2017; Hermann and Thaler, 2021) have described only a few fully identified compounds that serve as cues for predatory search and attack on live, intact prey. Within ocean habitats, dimethylsulfoniopropionate (DMSP) and dimethylsulfide (DMS) mediate multitrophic interactions among primary producers (phytoplankton, Dacey and Wakeham, 1986), herbivores (zooplankton, Steinke et al., 2006), higher-order consumers (Procellariiform birds, Nevitt et al., 1995; Savoca and Nevitt, 2014) and biodegradatory bacteria (Zimmer-Faust et al., 1996; Seymour et al., 2010). Additional attractants include waterborne toxins and secondary metabolites, such as tetrodotoxin, tamjamines A and B, halimedatetraacetate and 4-hydroxybenzoic acid (Carté and Faulkner, 1986; Gavagnin et al., 2000; Hwang et al., 2007; Bornancin et al., 2017). Upon their release from chemically defended prey, these compounds evoke searching and feeding responses in resistant and tolerant predatory species.

In the aquatic domain, substantial attention has been directed towards free-amino acids, quaternary ammonium bases, nucleotides and organic acids as feeding attractants and stimulants (Carr et al., 1984; McClintock et al., 1984; Valentinčič, 1985; Zimmer-Faust, 1993; Luis and Gago, 2021; Teoh et al., 2023). These low molecular weight compounds are found in abundance within the flesh of both fish and invertebrate prey (Carr et al., 1996) and have served as valuable tools in linking appetitive feeding to the underlying chemosensory mechanisms (Caprio, 1978; Zimmer-Faust et al., 1984; Derby and Ache, 1984; Carr and Derby, 1986; Atema et al., 1989; Valentinčič et al., 2000). Spiny lobsters, for example, can be trained to discriminate among complex mixtures derived from various flesh sources (crab, shrimp, mullet and oyster) based solely on differences in blend ratios (Fine-Levy et al., 1989). However, these identified free-amino acids, quaternary ammonium bases and nucleotides are not emitted in abundance, or at all, by live, intact prey; instead, they are degraded to metabolites and released as waste following dietary ingestion or uptake from seawater (Manahan et al., 1982; Derby and Zimmer, 2012).

New research is sorely needed on the chemical basis for live prey recognition, especially by aquatic consumers. To our knowledge, no published work has yet revealed the complete chemical identities of the molecules that guide generalist carnivore preferences for different prey species in either aquatic or terrestrial habitats. Familiarity with the identities and fundamental properties of these attractants and stimulants could yield valuable insights into the sensory mechanisms of resource exploitation.

### Study system

In light of these research imperatives, we explored the chemosensory basis for predator–prey interactions along wave-swept shores. Here, hard-shelled mussels (*Mytilus californianus*) and barnacles (*Balanus glandula*) not only function as foundation species, but also constitute abundant prey (Trussel et al., 2003; Robles et al., 2021). The mussels and barnacles secrete either a 29.6 kDa (named ‘KEYSTONEin’) or a 199.6 kDa (named ‘MULTIFUNCin’) glycoprotein, respectively, with expression confined to their body armors (epidermis, cuticle, live shell materials) (Ferrier et al., 2016a; Zimmer et al., 2017). Consequently, KEYSTONEin and MULTIFUNCin are available for contact recognition by predators that traverse rocky substrates in their pursuit of live, intact prey.

Sea stars (*Pisaster ochraceus*) and whelks (*Acanthinucella spirata*, a marine snail) are generalist carnivores and principal consumers of both mussels and barnacles in rocky, intertidal habitats (Feder, 1959; Paine, 1966; 1974; Mauzey et al., 1968; Murdoch, 1969; Dayton, 1971). Whereas whelks possess contact chemoreceptors that are diffusely distributed across their foot and concentrated on a retractable proboscis (Crisp, 1971; Hodgson and Brown, 1985), sea stars explore the substrate with contact chemoreceptors located on their tube feet (McClintock et al., 1994). In fact, KEYSTONEin and MULTIFUNCin are each necessary and sufficient to induce predatory attacks and feeding on live, intact mussels (Zimmer et al., 2016b, 2017) and barnacles (Ferrier et al., 2016a,b; Zimmer et al., 2016a, 2021).

These initial discoveries suggest several additional questions. Do sea stars and whelks have similar or distinct chemical preferences, when offered a choice? If they do differ, does one of these consumer species prefer KEYSTONEin while the other favors MULTIFUNCin? And if there are distinctions, can KEYSTONEin and MULTIFUNCin alone adequately explain the observed prey preferences? Furthermore, what are the respective roles of chemoreception and mechanoreception (relating to prey shell architecture) in influencing prey selection? The present study addresses and experimentally answers these questions using faux mussels and barnacles designed to simulate essential physical and chemical characteristics of their live, intact counterparts. Here, we show that prey preferences are guided by singular, glycoprotein cues without the need for low molecular weight stimulants or quality coding via mixture blends.

## MATERIALS AND METHODS

### Isolation and purification of bioactive proteins

Chemical fractionations, isolations and purifications of MULTIFUNCin and KEYSTONEin followed established procedures (Ferrier et al., 2016a; Zimmer et al., 2016b, 2017). Purity and concentration of each compound were confirmed using matrix-assisted laser desorption ionization time-of-flight mass spectrometry (Voyager DE-STR, Applied Biosystems, Foster City, CA, USA). Elsewhere, translations of complete amino acid and nucleotide sequences were described for each molecule and its encoding gene (GenBank accession numbers: KC152471 for MULTIFUNCin, and KC152469 for KEYSTONEin).

#### MULTIFUNCin

Live barnacles (*Balanus glandula* Darwin 1854) (0.4 to 1.0 cm shell height) were collected in Malibu, CA, USA, flash-frozen on-site in liquid nitrogen, and transported directly to our UCLA laboratory. Here, individuals were combined, crushed and homogenized (with shells) at high speed (using a Waring blender) in a 1:1.5 (v/v) mixture of 50 mmol l^−1^ trishydroxymethylaminomethane-HCl (hereafter, tris-HCl) buffer at pH 7.5. Resulting homogenates were stirred on ice for 120 min, rough filtered through gauze (80 µm nominal pore diameter), and centrifuged at 40,000 ***g*** (for 30 min at 4°C). Supernatants were filtered to 0.45 µm, and eluates were retained as crude extracts.

Initial purification steps involved separating eluate components on the basis of solubility through stepwise ammonium sulfate [(NH_4_)_2_SO_4_] precipitation. Bioactive proteins were isolated from other, organic molecules starting with 35%, increasing to 70% and ending with 100% (NH_4_)_2_SO_4_ saturation. Ammonium sulfate was added to each crude extract and stirred at 4°C for 10 min, then centrifuged at 40,000 ***g*** (4°C for 15 min), before the supernatant was removed. MULTIFUNCin was retained in precipitate of the 35 to 70% saturation solution (Ferrier et al., 2016a). Each pellet was resuspended in 2 ml of 50 mmol l^−1^ tris-HCl (pH 7.5).

Resuspended precipitates were fractionated using size-exclusion (SEC) and lentil-lectin (LCA) affinity chromatography. A HiPrep 16/60 high resolution S-200 sephacryl gel size-exclusion column (SEC, GE Healthcare, Waukesha, WI, USA) was eluted at 0.8 ml min^−1^ with 50 mmol l^−1^ tris-HCl (pH 7.5) buffer on a Biologic^TM^ fast performance liquid chromatography (FPLC) system (Bio-Rad, Hercules, CA, USA). MULTIFUNCin was retained in the 150 to 530 kDa SEC peak (Ferrier et al., 2016a). Each peak was collected, and divided into two samples based on the presence/absence of glycan groups. A 16/40 column (GE Healthcare) was packed with LCA sepharose 4B beads (GE Healthcare), and equilibrated with an FPLC system running buffer (0.5 mol l^−1^ NaCl, 25 mmol l^−1^ tris-HCl, pH 7.5) at 0.8 ml min^−1^. Concentrated (1 mg ml^−1^) 150–530 kDa peak material was washed through the LCA beads with an additional 150 ml of equilibrated buffer. MULTIFUNCin, bound to the LCA beads following the wash, was removed with 150 ml of elution buffer (0.2 mol l^−1^ methyl α,D-mannopyranoside, 0.5 mol l^−1^ NaCl, 25 mmol l^−1^ tris-HCl, pH 7.5) increasing linearly from 0 to 100% over 30 s at the same flow rate. It was then concentrated using ultrafiltration [molecular weight cut-off (MWCO)=10 kDa].

Final purification involved protein separation and isolation through preparative SDS-PAGE. Each sample (1 ml) containing MULTIFUNCin was conditioned with 5× Laemmli buffer and loaded onto a 7.5% acrylamide (acrylamide:bis=29:1) slab gel (16×22 cm). It was run on ice at 180 V, until clear separation of molecular weight markers. All protein bands were visualized with imidazole-zinc reverse staining. From each gel, the MULTIFUNCin band was excised with a sterile scalpel, de-stained in Laemmli buffer, and recovered from the acrylamide via electroelution. Each gel fragment containing MULTIFUNCin was put into a Spectra/Por regenerated cellulose dialysis tubing (1 kDa MWCO, 8 ml volume; Spectrum Medical Corp., Long Beach, CA, USA), sealed and placed between the electrodes of a western blotting rig. Both tubing and rig were filled with Laemmli buffer, stirred and cooled on ice. An electric current (100 V) was applied for 1 h. Isolated MULTIFUNCin migrated out of each fragment and into the buffer. Following electroelution, selected fragments were removed and stained with Coomassie G-250 dye to check protein recovery (which averaged ∼65%). The buffers were exchanged, through dialysis, to 50 mmol l^−1^ tris-HCl (pH 7.5). Purified MULTIFUNCin was concentrated 10-fold through ultrafiltration (MWCO=10 kDa), dialyzed (Slide-A-Lyzer^TM^ dialysis cassettes, 5 kDa MWCO, Thermo Fisher Scientific, Waltham, MA, USA) and diluted with 0.45 µm-filtered seawater, as required for bioassay. When not used immediately in bioassays, MULTIFUNCin was stored at −80°C in 50 mmol l^−1^ tris-HCl (pH 7.5).

#### KEYSTONEin

Like barnacles, live mussels (*Mytilus californianus* Conrad 1837) (1.5–4.0 cm shell length) were collected in Malibu, CA, USA, placed on ice, and transported directly to our laboratory. Mussel extrapallial fluid is a major source of KEYSTONEin. This compound is secreted by the mantle epidermis prior to incorporation into shell materials (Zimmer et al., 2017). Extrapallial fluid (0.1–1.0 ml per mussel) was carefully removed by catheter without damaging tissues. Immediately following collection, bioactive material in each 10 ml (combined) batch was isolated from impurities starting with 25%, increasing to 50%, then 75%, and ending with 100% NH_4_(SO_4_)_2_ saturation. KEYSTONEin was retained as precipitate in the 50–75% saturation solution (Zimmer et al., 2016b, 2017). The KEYSTONEin-containing precipitate was resuspended in 50 mmol l^−1^ tris-HCl buffer (pH 7.5). It was then subjected to preparative SDS-PAGE and electroelution, using identical procedures as described above for MULTIFUNCin. Further purification was unnecessary by means of SEC or LCA affinity chromatography. Recovery of isolated KEYSTONEin from select SDS-PAGE gel fragments averaged ∼75%. Fully purified material was resuspended through dialysis in 0.45 µm-filtered seawater as needed for bioassays. When not used immediately in bioassays, KEYSTONEin was stored at −80°C in 50 mmol l^−1^ tris-HCl (pH 7.5).

### The chemical basis for prey preference: faux prey

Faux prey were constructed to mimic meaningful physical and chemical characteristics of live barnacles and mussels. In each experiment, all soft tissues, cuticles and biological fluids were removed with sterile instruments from live barnacles [0.4–0.6 cm shell heights for experiments on whelks, *Acanthinucella spirata* (Blainville 1832); 0.8–1.0 shell heights for sea stars, *Pisaster ochraceus* (Brandt 1835)] and mussels (1.5–2.5 cm shell lengths for experiments on whelks; 3.0–4.0 cm shell lengths for sea stars). Mussels are naturally larger than barnacles, and chosen size classes were those preferred by sea stars and whelks in feeding trials (McClintock and Robnett, 1986; Ferrier, 2010). The empty shells were combusted for 12 h at 150°C, bathed for 15 min in a stirred, 5% (v/v) HCl solution (in tris-HCl buffer), and scrubbed thoroughly with Nanopure-grade deionized water to eliminate any residual organics. Cleaned, empty barnacle and mussel shells served as physical replicas (‘faux’) of the live animals. To a human observer, these models were indistinguishable in color and texture from the live, intact animals.

Prey chemistry was simulated in protein-laced gels. Sodium carboxymethylcellulose (11.7% w/v) was mixed with 0.45-µm filtered seawater and either purified MULTIFUNCin or KEYSTONEin, then cured for 10 min at 4°C. Once prepared, each gel had the consistency of prey flesh and was injected into a cleaned, empty barnacle or mussel shell for immediate use in experiments. For each faux mussel, shell valves were held together using a rubber band. Gel protruded 1 mm beyond the shell margin to simulate the mantle of a live animal. Concentrations of MULTIFUNCin (25 µg ml^−1^, ∼1.2×10^−7^ mol l^−1^) and KEYSTONEin (70 µg ml^−1^, ∼2.5×10^−6^ mol l^−1^) were carefully matched between gels and live shell materials. From repeated chemical measurements, there was no evidence for photo-oxidation or biodegradation of the bioactive compounds during preparatory and isolation procedures, or in bioassay experiments.

### Animal maintenance and holding procedures

Bioassays were performed during July through September, when sea stars and whelks feed most actively in native habitats. Both species are non-visual predators and graze solitarily on rocky, wave-swept shores. Following collections in Malibu, animals were brought to the lab, fed an *ad libitum* diet for 1–2 weeks, then fasted for 3–5 days before testing. A combination of barnacles and mussels were maintained abundantly in each tank. Feeding by whelks and sea stars was observed on most days. Of those predators eating prey during holding, ≥85% of the time whelks ate barnacles whereas sea stars ate mussels. The lab setup consisted of a 28,000 l reservoir of seawater (oceanic quality, 33 psu salinity; Catalina Water LLC, Long Beach, CA, USA), with particle filtration (5 µm cut-off), UV sterilization and computer-controlled water/air temperature (±1°C off set-points) and light cycle (14 h:10 h light:dark, lights on at 06:30 h). During animal holding periods and experiments, water temperature was maintained at 19–20°C and lighting (full spectrum, General Electric Daylight Ultra) was held at a level (75 µmol m^−2^ s^−1^) simulating field habitats during late afternoon. Each species was kept separately at a density of 12–15 m^−2^ (whelks) or 3–4 m^−2^ (sea stars) in continuously recirculating seawater (45 l min^−1^), replenished at least twice per week. Whelks were 1.5–2.5 cm in shell length and sea stars 7–12 cm in arm length (measured from the proximal tip to the center of the oral disc).

### General experimental procedures

All bioassays were performed in Plexiglas arenas. These tanks were scaled to predator size: 60×60×15 cm (length×width×depth) for sea stars, or 10×10×5 cm for whelks. A constant supply of single-pass, 5-µm filtered seawater (1 l min^−1^) was delivered to every arena, held at ambient ocean temperature and salinity (see ‘Animal maintenance and holding procedures’ above). Each tank was washed thoroughly with 1% (v/v) HCl in Nanopure water after every trial, and then rinsed with Nanopure water and seawater to eliminate any residual organics. All trials were performed between 10:00 and 18:00 h.

We conducted a total of four sets of experiments to ascertain feeding preferences of whelks (*A. spirata*) and sea stars (*P. ochraceus*). A minimum of 16 replicate trials, each using a different predatory animal, was performed per experiment. Each trial involved one or more groups of treated faux prey surrounding one consumer. A trial began with the introduction of a predator at the center of an arena. It ended when this consumer selected and ate a single faux prey, or was terminated after 1 h regardless. The treatments consisted of faux prey laden with either KEYSTONEin, MULTIFUNCin or seawater (control) infused gel, replaced after every trial. The exact positioning of each treatment within a group was determined using a random number generator, and was changed from trial to trial. Two similar experiments, one with whelks and one with sea stars, comprised a set.

### Preference experiments

**Fig. 1.**
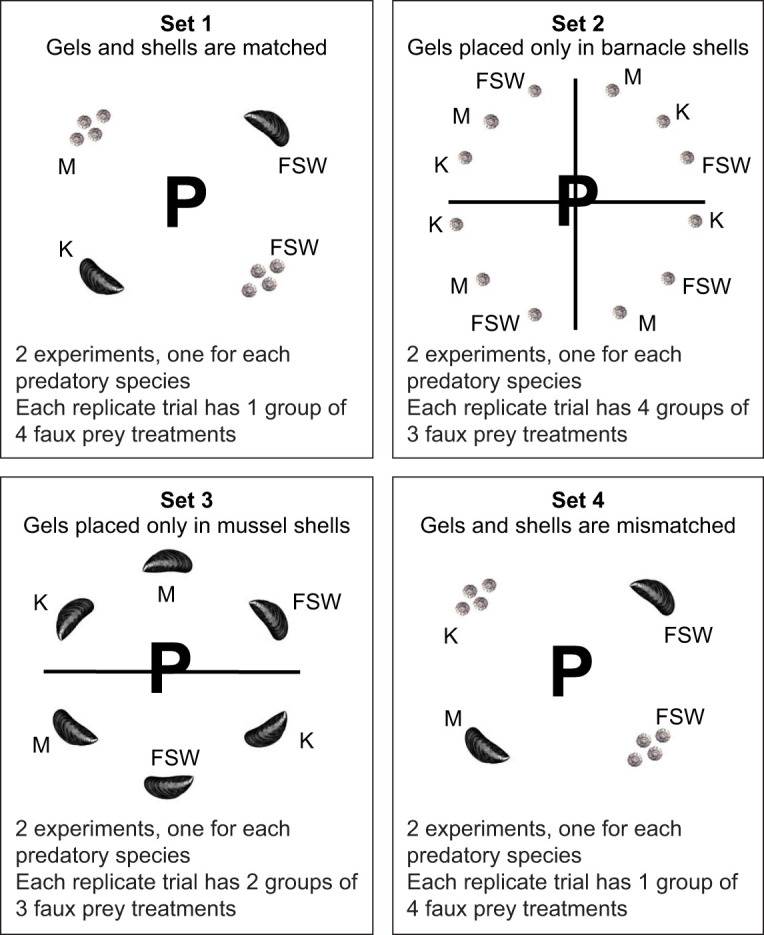
**A schematic depicting set 1, 2, 3 and 4 experiments.** Images are not drawn to scale. Each picture of a single barnacle (*Balanus glandula*) or mussel (*Mytilus californianus*) represents a clean, empty shell with either MULTIFUNCin (M), KEYSTONEin (K) or filtered seawater (FSW) infused gel. Two similar experiments were performed in each set for whelks (*Acanthinucella spirata*) and sea stars (*Pisaster ochraceus*). Only one predator (P) was placed per trial into an arena, and never reused. Faux prey were positioned equidistantly from one another, surrounding the centrally located predator. The exact position of each faux prey treatment within a group was chosen using a random numbers table, and the randomized order changed from trial to trial. A trial began when a predator was first introduced, and was stopped when this consumer ate a single faux prey, or after 1 h without feeding. To compensate for size differences in sets 1 and 4, we placed 4–7 faux barnacles for each faux mussel. This procedure ensured that both prey types covered approximately the same bottom surface area of each arena. A minimum of 16 trials was conducted per experiment. Each trial employed one group with four faux-prey treatments in sets 1 and 4; four groups, each with three treatments in set 2; and two groups, each with three treatments in set 3. All other details pertaining to experimental trials and treatments are provided in the text (see Materials and Methods, Preference tests).

The initial set (set 1) addressed two questions: (1) do whelks and sea stars prefer the same or different prey species, and (2) is MULTIFUNCin or KEYSTONEin required for prey recognition? The experimental treatments using faux prey (shell type and gel chemistry) were designed to simulate nature. Each faux prey contained one of four treatments, and they encircled the predator during a trial (Fig. 1A). Treatment A featured a single faux mussel shell filled with KEYSTONEin gel; treatment B had a single faux mussel shell containing seawater gel; treatment C consisted of 4–7 faux barnacle shells, each infused with MULTIFUNCin gel; and treatment D comprised 4–7 faux barnacle shells, each containing seawater gel. Because a single faux mussel covered a larger bottom surface area in the test arena compared with a single faux barnacle, a mussel was more likely to be encountered at random by searching whelks or sea stars. To address this potential bias, we scaled the number of faux barnacles to ensure that both prey types covered approximately the same bottom area.

Experiments in sets 2, 3 and 4 were designed to elucidate the dominant sensory modality in prey selection, whether it be chemoreception (faux prey chemistry) or mechanoreception (faux prey shell morphology). Sets 2 and 3 addressed the question: do whelks and sea stars prefer the same, or a different, compound (MULTIFUNCin or KEYSTONEin) when shell-type is held constant? In set 2, faux prey were divided into four groups, each consisting of three treatments (Fig. 1B). In each treatment, a single faux barnacle shell was filled with either MULTIFUNCin (treatment A), KEYSTONEin (treatment B) or filtered seawater (treatment C). Set 3 featured faux prey arranged in two groups, each with three treatments (Fig. 1C). In this case, each treatment comprised a single faux mussel shell infused with either KEYSTONEin (treatment A), MULTIFUNCin (treatment B) or filtered seawater (treatment C). Set 4 considered the question: do whelks and sea stars rely on chemistry alone, or on both chemical and physical attributes when choosing prey? Shell type and gel chemistry were deliberately mismatched within each faux prey. Similar to set 1 experiments, faux prey items were arranged into four treatments encircling the predator (Fig. 1D). Now, however, treatment A consisted of a single faux mussel shell filled with MULTIFUNCin-infused gel; treatment B had a single faux mussel shell containing seawater-impregnated gel; treatment C included 4–7 faux barnacle shells, each with KEYSTONEin gel; and treatment D comprised 4–7 faux barnacle shells, each imbued with seawater gel. The placement of each treatment within a group was determined using a random number generator, as previously described.

## RESULTS

Sea stars (*P. ochraceus*) and whelks (*A. spirata*) are major consumers of mussels (*M. californianus*) and barnacles (*B. glandula*) in their native habitats (Murdoch, 1969; Paine, 1974; Ferrier, 2010). Previous field and laboratory studies have established that barnacle MULTIFUNCin and mussel KEYSTONEin are each necessary and sufficient to trigger attack and feeding responses in both predatory species (Ferrier et al., 2016a,b; Zimmer et al., 2016a, 2017, 2021). These prior experiments, however, did not test for preferences by sea stars and whelks between KEYSTONEin and MULTIFUNCin. In the present study, we used gels infused into cleaned natural shells to create faux prey, allowing us to determine, experimentally, the contact-chemosensory basis for prey preferences. The ability to isolate physical (shells) from chemical (MULTIFUNCin, KEYSTONEin) prey properties proved to be a valuable tool revealing predator choices in these experiments.

Whelks and sea stars showed positive responses by feeding on faux prey in 55–61% or 55–65%, respectively, of all replicate trials in each of our eight experiments. The responsiveness of whelks and sea stars in the laboratory thus mirrored those observed in repeated field surveys at the Malibu collection sites. In the field, 54–72% of all censused whelks and 55–62% of all censused sea stars were found feeding daily on either barnacles or mussels, respectively, during summer months (July–September) (Ferrier, 2010; Ali, 2011). Therefore, our current laboratory bioassay results reflect findings from native environments.

**Fig. 2.**
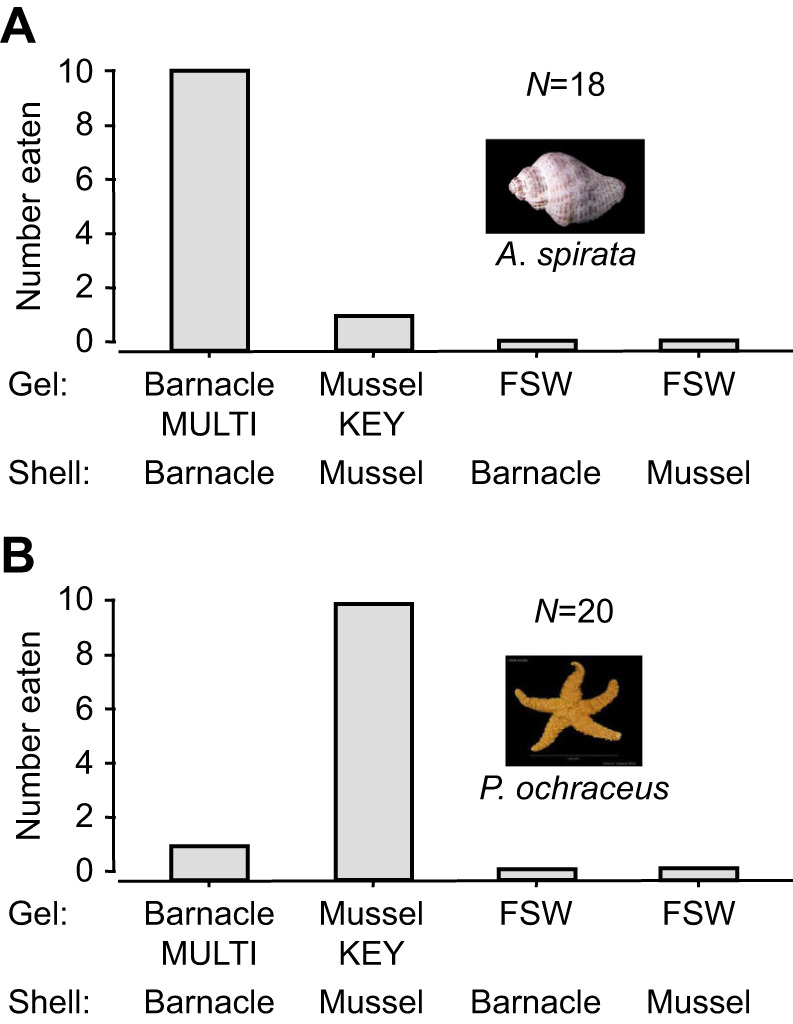
**Feeding preferences of whelks and sea stars in set 1 experiments.** (A) Whelks; (B) sea stars. Here, we matched shell type with gel chemistry for each faux prey species. Each predator was allowed to consume no more than one faux prey per trial. As shown in A, there are 18 total replicate trials (*N*); a whelk ate one of the four faux prey treatments (10+1+0+0) in 11 trials, and a whelk failed to feed in 18–11=7 trials. For each experiment, a bar depicts the total number of replicate trials in which a given faux prey treatment was eaten. MULTI, MULTIFUNCin-infused gels; KEY, KEYSTONEin-infused gels; FSW, 0.45 µm-filtered seawater infused gels.

In set 1 experiments, we matched gel chemistry with shell type for each prey species. Whelks fed significantly more on barnacle faux prey, as opposed to mussel or seawater faux prey [sign test with Bonferroni's correction (α=0.05/6=0.008): *P*<0.0058, for both comparisons] (Fig. 2A). Conversely, sea stars largely ignored barnacle and seawater faux prey, showing a strong preference for mussel faux prey infused with KEYSTONEin (sign test with Bonferroni's correction: *P*<0.0058, for both comparisons) (Fig. 2B). Therefore, each consumer species exhibited a clear and contrasting prey preference, with either MULTIFUNCin or KEYSTONEin required to induce a predatory response.

**Fig. 3.**
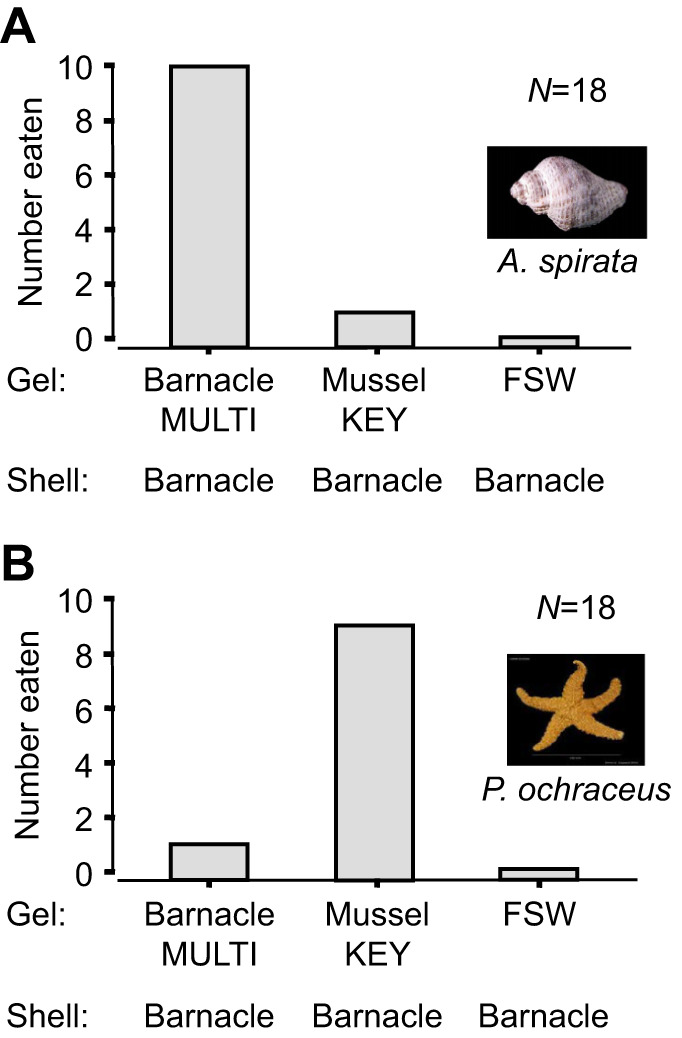
**Feeding preferences of whelks and sea stars in set 2 experiments.** (A) Whelks; (B) sea stars. Here, we presented only protein- or seawater-laced gels within cleaned barnacle shells. All other conditions are as described in the Fig. 2 legend.

**Fig. 4.**
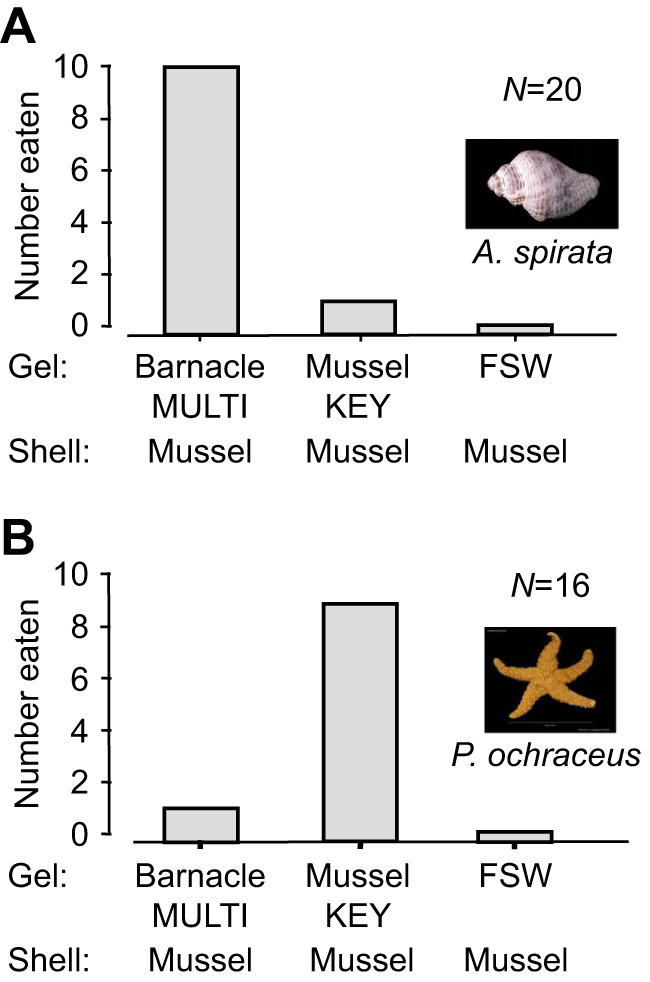
**Feeding preferences of whelks and sea stars in set 3 experiments.** (A) Whelks; (B) sea stars. Here, we presented only protein- or seawater-laced gels within cleaned mussel shells. All other conditions are as described in the Fig. 2 and 3 legends.

The experiments in sets 2 and 3 were designed to assess the relative impact of prey chemistry on prey preferences. In both sets, whelks and sea stars consistently demonstrated a strong preference for either MULTIFUNCin- or KEYSTONEin-laced gel, regardless of the shell type, or other gel treatments [sign test with Bonferroni's correction (α=0.05/3=0.017): *P*<0.0058, all comparisons] (Figs 3 and 4). These findings underscore the dominance of specific molecular properties of each compound, indicating different chemical preferences between sea stars and whelks.

**Fig. 5.**
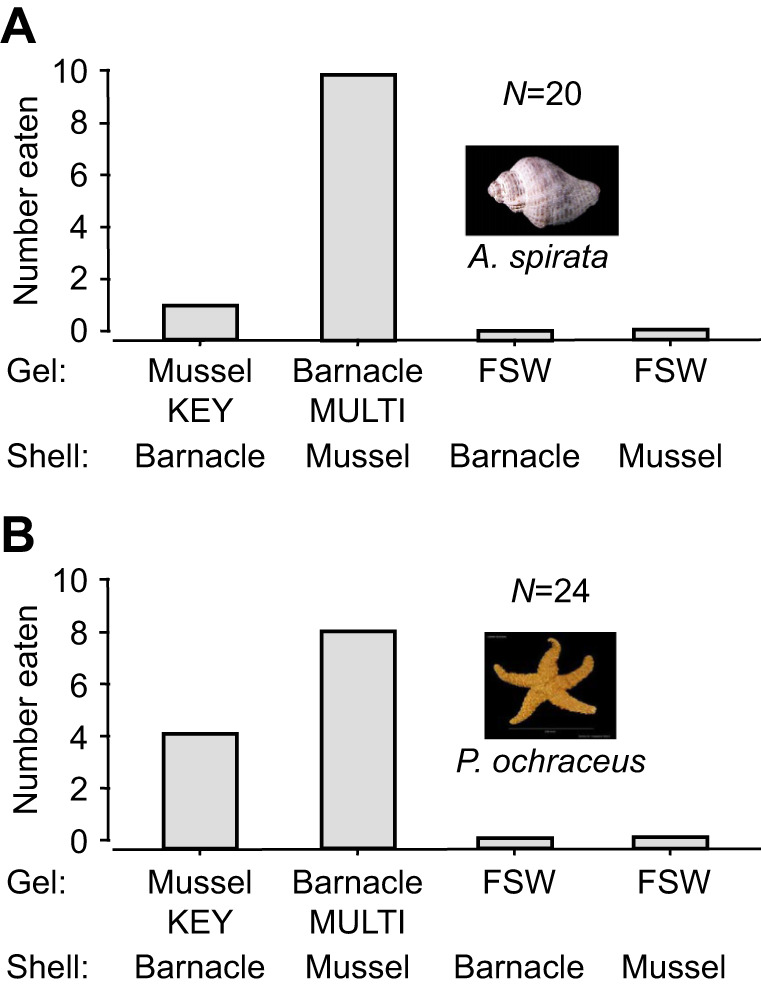
**Feeding preferences of whelks and sea stars in set 4 experiments.** (A) Whelks; (B) sea stars. Here, we deliberately mismatched shell type with gel chemistry for each faux prey. All other conditions are as described in the Fig. 2 and 3 legends.

The set 4 experiments created a more challenging scenario for predatory whelks and sea stars by deliberately mismatching the gel chemistry with the shell type in each treatment. The purpose was to assess more definitively the relative contributions of prey chemistry versus physical attributes in determining predator preferences. As in sets 1, 2 and 3, we found that whelks significantly favored MULTIFUNCin over KEYSTONEin and seawater (sign test with Bonferroni's correction: *P*<0.0058, in all comparisons) (Fig. 5A). Whelks were not swayed by the mismatches in set 4, and continued to attack and feed based solely on their preferred prey chemistry, with no detectable influence from shell type. In contrast, sea stars exhibited a different response. When prey stimuli were mismatched by shell type, sea stars showed a partial preference for the MULTIFUNCin/mussel–shell treatment combination over the KEYSTONEin/barnacle–shell combination (Fig. 5B) (sign test with Bonferroni's correction: *P*=0.194). Thus, unlike whelks, the decision-making process of sea stars was influenced by an interaction between chemical properties and physical attributes of the prey.

## DISCUSSION

### Chemosensory mechanisms guiding prey preferences

Studies on carnivorous animals have long considered the effects of prey-associated chemical cues, classifying them as attractants, deterrents, stimulants or suppressants. The underlying chemosensory basis for consumer searching and feeding has been especially well described in terrestrial organisms (Ferrero et al., 2011; Apfelbach et al., 2015). For several predatory species, windborne or substrate-adsorbed molecules have been accurately identified as cues to the presence of live, intact prey (Raffa et al., 2007; Smargiassi et al., 2012; Xue et al., 2018). In contrast to their terrestrial counterparts, relatively little is known about the chemistry of live, intact prey in aquatic environments. Although researchers have explored the chemosensory mechanisms guiding searching and feeding in aquatic consumers, the majority of this work has focused on low molecular weight molecules found in excised flesh that are seldom, or not at all, released by live, intact prey (Derby and Zimmer, 2012). To our understanding, no published work has yet revealed the complete structural identities of the molecules that establish preferences by generalist carnivores for different prey species in either the aquatic or terrestrial realm. This dearth of knowledge on chemosensory mechanisms persists despite considerable efforts by behavioral biologists and ecologists to determine the nature of carnivore prey preferences (see literature citations for Chesson, 1978, 1983, and Pearre, 1982, in Web of Science and Google Scholar).

The present bioassays, using two generalist predatory species and two purified (MULTIFUNCin and KEYSTONEin) glycoprotein cues, have linked carnivore preferences to the chemistries of their live, intact prey species. Whelks and sea stars were selected for the present study owing to their critical ecological significance and their representation across the protostome (mollusks)–deuterostome (echinoderms) division. After 670 million years of genetic isolation (Ayala et al., 1998), molecular mechanisms for chemical recognition differ considerably among protostomes and deuterostomes (Bargmann, 2006; Cummins et al., 2009; Derby et al., 2016). Yet, for sea star and whelk consumers, glycoprotein detection has converged across the divide to promote predation on valuable shared prey resources.

Distinctions in contact-chemoreception between *A. spirata* and *P. ochraceus* became apparent only after KEYSTONEin- and MULTIFUNCin-infused faux prey were tested, simultaneously, in our current preference tests. When these carnivores were given a choice in the set 1 experiments, simulating nature, whelks showed a strong preference for MULTIFUNCin in barnacle shells, and sea stars for KEYSTONEin in mussel shells. These unique chemical and physical characteristics of the faux prey species can thus explain the inherent feeding preferences of sea stars for live mussels over barnacles (Mauzey et al., 1968) and whelks for barnacles over mussels (Ferrier, 2010) in their native habitats. The fidelity of the glycoprotein cues was furthermore illustrated in experiments controlling for shell type (sets 2 and 3), where disparities in chemistry alone dictated the faux prey preferences of sea stars versus whelks.

The sensory mechanisms governing prey preferences in sea stars and whelks diverged in the set 4 experiments, which intentionally mismatched gel flavor and shell type. Notably, whelks displayed no discernible response to shell type and exhibited a strong penchant for MULTIFUNCin-flavored gels, regardless of its location (barnacle or mussel shells). In contrast, sea stars showed preferences shaped by both the physical and chemical properties of faux prey. Some individuals opted for smaller faux prey (barnacle shells) containing the preferred chemistry (KEYSTONEin), whereas other individuals chose larger faux prey (mussel shells) imbued with MULTIFUNCin. In this case, a stronger response by sea stars to the larger prey may reflect a higher perceived energetic reward (McClintock and Robnett, 1986).

### Linking carnivore preferences to environmental settings and contact-chemosensory information

Natural ecological communities are characterized by intricate arrays of species assemblages (Morin, 2009). Similar to sea stars and whelks, the majority of predatory species within these communities are generalists, consuming a variety of prey taxa. When a carnivore targets a specific prey, it does so amidst a multitude of sensory cues emanating from a diverse assortment of organisms. To mitigate these complicating factors, laboratory studies on the molecular identities of contact-chemical cues have often taken a highly reductionist approach. These investigations most commonly have focused on interactions between single predator and single prey species, usually in isolated experimental settings devoid of extraneous stimuli (Skelhorn and Rowe, 2006; Xue et al., 2018; Humbel et al., 2021). Such research has commonly employed bioassays to assess predatory feeding responses to chemical fractions, mixtures or single compounds derived from prey, applied to filter paper or other types of artificial substrates (Smargiassi et al., 2012; Binz et al., 2016; van Giesen et al., 2020; Humbel et al., 2021). Over time, these reductionist approaches have successfully identified bioactive molecules, especially amongst terrestrial organisms (Smargiassi et al., 2012; Binz et al., 2016). They have underscored the necessity of specific compounds or compound mixtures to evoke predatory responses upon contact. However, they have not adequately addressed whether these compounds alone are necessary and sufficient to induce predation on intact prey. Furthermore, it remains uncertain how these isolated bioactive molecules would have competed for a carnivore's attention in a more natural and intricate sensory environment.

Using realistic faux prey in bioassays overcomes several limitations of previous studies. When carefully crafted, these models closely emulate the appearances and textures of live prey (Lang and Menzel, 2011; Saviola et al., 2013; Xue et al., 2018; present study). The chemical composition can be manipulated and adjusted while largely preserving the natural tactile and visual cues. Additionally, faux prey can be deployed in both laboratory and field settings. This latter approach facilitates controlled bioassay experiments amidst a variety of sensory cues from the diverse assemblages of organisms present in natural habitats (Zimmer et al., 2016a, 2017). Simultaneously deploying faux prey from two or more species enhances realism in bioassays, particularly in studies involving generalist predators and revealing their chemical preferences. Moreover, experimental treatments that mismatch physical and chemical prey attributes enable the determination of whether a particular compound (or mixture) alone is necessary and sufficient to induce predatory attack.

Thousands of metazoan prey species, ranging from marine sponges to terrestrial vertebrates, secrete glycoprotein-rich extracellular products similar to those of mussels and barnacles (Sarashina and Endo, 2006; Ehrlich, 2010; Wang and Nilson-Hamilton, 2013). Predators, whether terrestrial or aquatic, have evolved to exploit these surface-adsorbed compounds as contact-chemosensory cues (Leroy et al., 2006; Smargiassi et al., 2012; Saviola et al., 2013; Zimmer et al., 2021). However, previous investigations on protein cues were limited to single predator–single prey species interactions. The present study, in contrast, illustrates that consumer preferences among different prey species can hinge on only one or two isolated contact-protein cues, without requiring additional chemosensory inputs. Moreover, it underscores that predator preferences for prey can shift depending on the unique environmental context in which they encounter these contact-chemical stimuli.
